# Sonodynamic-immunomodulatory nanostimulators activate pyroptosis and remodel tumor microenvironment for enhanced tumor immunotherapy

**DOI:** 10.7150/thno.79945

**Published:** 2023-03-05

**Authors:** Zhuang Chen, Weijing Liu, Zuo Yang, Yi Luo, Chaoqiang Qiao, Anna Xie, Qian Jia, Peng Yang, Zhongliang Wang, Ruili Zhang

**Affiliations:** 1 Lab of Molecular Imaging and Translational Medicine (MITM), Engineering Research Center of Molecular and Neuro Imaging, Ministry of Education, School of Life Science and Technology, Xidian University & International Joint Research Center for Advanced Medical Imaging and Intelligent Diagnosis and Treatment, Xi'an, Shaanxi, 710126, P. R. China; 2 Academy of Advanced Interdisciplinary Research, Xidian University, Xi'an, Shaanxi, 710071, China.

**Keywords:** pyroptosis, gasdermin E, sonodynamic therapy, tumor microenvironment normalization

## Abstract

**Rationale:** Spatiotemporal control of pyroptosis has a profound impact on cancer immunotherapy. Owing to the precise spatiotemporal control and reduction in the side effects of ultrasound (US), sonodynamic therapy (SDT) is expected to be a promising mean to activate pyroptosis. Furthermore, the pyroptosis-initiated immune response can be amplified by enhanced lymphocyte infiltration occurring due to extracellular matrix (ECM) depletion. Therefore, it is highly desirable to develop a sonodynamic-immunomodulatory strategy to amplify pyroptosis-mediated tumor immunotherapy by remodeling of the tumor microenvironment, thereby enhancing tumor immunotherapy.

**Methods:** We reported a potent strategy based on a sonosensitizer, which is composed of LY364947-loaded porous coordination network (PCN-224) camouflaged with a red blood cell (RBC) membrane and evaluated pyroptosis activation, collagen depletion, immunocyte infiltration, and adaptive immune response during the pyroptosis-initiated immune response *in vitro* and *in vivo*.

**Results:** The sonosensitizer generated reactive oxygen species (ROS) under US irradiation and initiated the caspase-3 apoptotic signaling pathway, which is regarded as the key upstream activator of gasdermin E (GSDME)-mediated pyroptosis. During the subsequent anti-tumor immune response mediated by pyroptosis, LY364947 loosened the ECM structure via collagen depletion, resulting in enhanced T-lymphocyte infiltration and nearly complete eradication of tumors in a mouse model with the formation of immunological memory.

**Conclusion:** Our findings indicate that sonodynamic-immunomodulatory pyroptotic strategy exhibits robust anti-tumor immune efficacy as well as provides novel insights into the role of pyroptosis in cancer immunology.

## Introduction

Pyroptosis is a newly defined mode of programmed cell death characterized by cell swelling, membrane disruption, and release of cytoplasmic contents and pro-inflammatory cytokines 1, 2. Owing of its ability to activate the innate immune response, pyroptosis is promising in tumor immunotherapy 3. Since pyroptosis can be activated by caspase-3-mediated gasdermin E (GSDME) cleavage, numerous attempts have been made to induce pyroptosis using chemotherapeutic agents that lead to drug resistance and unwanted side effects 4-7. Emerging non-invasive light-triggered pyroptosis, represented by photodynamic therapy (PDT), has attracted increasing attention in recent years 8-11. However, owing to the poor light penetration depth, the application of light-triggered pyroptosis is limited to superficial tumors. Therefore, the demand for novel approaches triggering efficient and safe pyroptosis during tumor therapy has increased 12.

Sonodynamic therapy (SDT) utilizes sonosensitizers to generate highly cytotoxic reactive oxygen species (ROS) that kill tumor cells and elicit immune responses 13-15. Therefore, SDT intrinsically performs better than PDT owing to its superior nature, including greater safety, ease of operation, deeper tissue penetration, and lack of drug resistance 16, 17. With the development of low-intensity focused ultrasound (FUS) technology, SDT, in conjunction with its precise spatiotemporal control and reduced side effects, is expected to be promising in inducing pyroptosis for antigen release at tumor sites. However, most tumors establish an infiltrate-excluded immunosuppression microenvironment caused by a stiff extracellular matrix (ECM). As a result, there are relatively void of cytotoxic lymphocytes (CTLs) in the tumor core 18, 19, which severely counteracts SDT/pyroptosis-activated systematic immune responses. Therefore, developing a sonodynamic-immunomodulatory strategy to amplify pyroptosis-mediated tumor immunotherapy by remodeling of the tumor microenvironment is highly desirable.

**Scheme 1:**
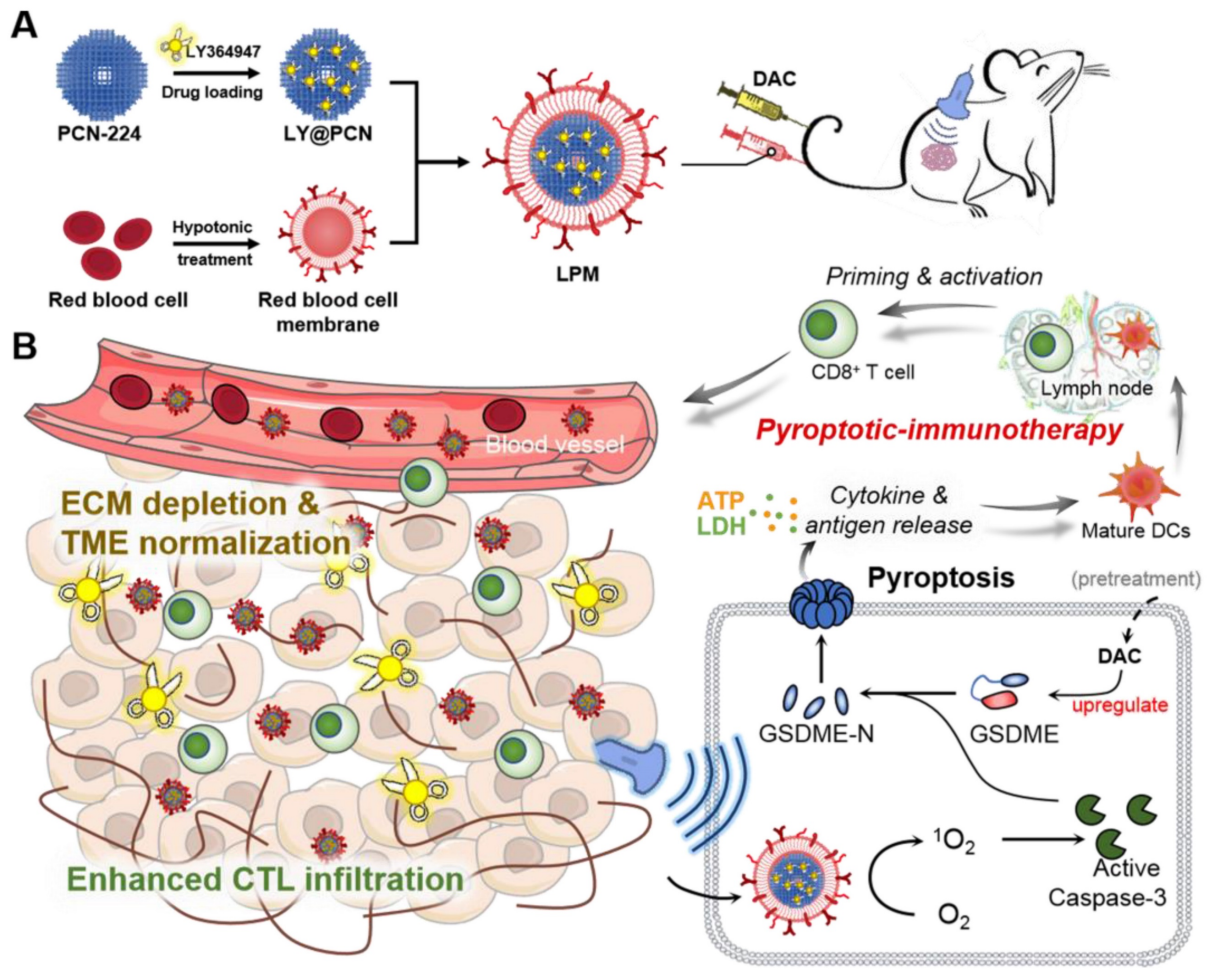
Schematic illustration of anti-tumor immunotherapy induced by the sonodynamic-immunomodulatory nanostimulator LPM. (A) Synthesis of LPM. (B) LPM are intravenously injected and accumulated in tumors. Upon ultrasound irradiation, plenty of reactive oxygen species produced and evoked pyroptosis of tumor cells, which can release tumor-associated antigen and inflammatory factors to stimulate DCs maturation. The presence of a dense extracellular matrix (ECM) in the tumor is a physical barrier that hinders the subsequent immune response. Released LY364947 induced ECM degradation, resulting in enhanced cytotoxic T lymphocytes infiltration and anti-tumor immunotherapeutic efficiency.

Herein, we demonstrated a combinational strategy to achieve enhanced cancer immunotherapy by SDT-induced pyroptosis and ECM normalization. Considering that the transforming growth factor-β type I (TGF-β1) plays an essential role in ECM formation as it stimulates the proliferation of fibroblasts and collagen deposition 20, blocking TGF-β1 receptor (TGFβRI) is supposed to effectively prevent collagen deposition and loosen the ECM structure. Therefore, LY364947 molecule, a selective inhibitor of TGFβRI, was chosen for ECM normalization 21, 22. However, low bioavailability, rapid clearance and poor targeting limit the application of LY364947 small molecule *in vivo*. Thus, porous coordination network (PCN-224) nanoparticles were selected as nanocarriers with sonosensitizer function for loading LY364947 and then further camouflaged with red blood cell (RBC) membranes for longer circulation and enhanced accumulation at the tumor site (Scheme 1A) 23-25. Upon ultrasound (US) irradiation, RBC membrane-coated PCN-224 nanoparticles (PM) generated robust ROS and induced caspase-3 activation, which further cleaved GSDME to initiate pyroptosis (Scheme 1B). To better demonstrate the effectiveness of our strategy, the expression of GSDME in tumor cells was upregulated with decitabine (DAC) pretreatment since GSDME expression in most tumor cells is silenced by methylation of DNA promoter. Along with collagen depletion and ECM normalization, LY364947 encapsulated PM (LPM) greatly enhanced T-lymphocyte infiltration during the subsequent pyroptosis-induced immune process. In a triple-negative breast cancer (TNBC) mouse model, LPM triggered considerable pyroptosis and eradication of subcutaneous tumor, more importantly proving the acquisition of anti-tumor adaptive immunity as a consequence of pyroptosis, thereby broadening the application of anti-tumor immunotherapies and providing novel insights into pyroptosis in cancer immunology.

## Materials and Methods

### Reagents and instrumentation

Zirconium oxychloride octahydrate (ZrOCl_2_·8H_2_O) was purchased from Innochem (Beijing) Technology Co., Ltd. Singlet oxygen sensor green (SOSG) was purchased from Thermo Fisher Scientific. DAC was purchased from Bide Pharmatech Ltd. The culture medium and fetal bovine serum were purchased from Gibco Life Technologies. The Western blot antibody was purchased from Abcam. Flow cytometry antibodies were purchased from Biolegend Ltd. Various kinds of kits were purchased from Beyotime Institute of Biotechnology Biotechnology. Solvents are available from general reagent®, including N, N-dimethylformamide (DMF), dimethyl sulfoxide (DMSO), and ethanol. Except as otherwise noted, all additional chemical reagents were bought from Sigma-Aldrich. The Animal Center of the Fourth Military Medical University (Xi'an, China) provided female BALB/c mice. In this study, animal protocols were reviewed and approved by the Fourth Military Medical University's Institutional Animal Care and Use Committee (approval number: 20190229).

### Synthesis of PCN-224

PCN-224 was synthesized in accordance with published papers 26. Briefly, DMF (7 mL), H_2_TCPP (5 mg), ZrOCl_2_·8H_2_O (15 mg), and benzoic acid (140 mg) were added into a stainless-steel autoclave lined with Teflon and heated to 90 °C for 5 h. As soon as the mixture cooled down to room temperature, centrifugation was performed, and fresh DMF and ethanol were used to wash the precipitate three times. The resulting PCN-224 nanoparticles were then suspended in ethanol for further use.

### Loading of LY364947 into PCN-224

In order to prepare LY364947-loaded PCN-224, a solution of LY364947 in DMSO was added dropwise into PCN-224 solution. Meanwhile, the mixture was treated with ultrasonic (45 W) for 30 min. After centrifugation at 12000 g for 10 min, the LY364947@PCN-224 nanoparticles (LP) were collected and washed twice with deionized water to remove the free LY364947.

### Extraction of red blood cell membrane

Red blood cell (RBC) membranes are prepared based on published protocol 27. In brief, the whole blood from female BALB/c mice was collected and stored in anti-coagulation tubes. First, the whole blood was centrifuged for 5 min at 800 g at 4 °C, and the supernatant with attached white blood cells were carefully removed. Afterward, the precipitates were washed with PBS until colorless supernatant appeared. Later, the sedimentary red blood cells were hemolyzed by 0.25 × PBS and then incubated at 4 °C for 2 h. To obtain the RBC membrane, the mixture was centrifuged and washed with 0.25% PBS solution three times. After being freeze-dried for 24 h, the RBC membrane was stored at -20 °C for further usage.

### Preparation of LPM

To prepare the RBC membrane-coated LP nanoparticles (LPM), the RBC membrane solution (5 mg mL^-1^) was made in 0.25 × PBS and added dropwise into the LP aqueous solution (2.5 mg mL^-1^), followed by sonication until the mixture became clear. Afterwards, the obtained LPM nanoparticles were centrifuged for 5 min at 13000 g and were washed three times with water. In addition, zeta potential, SDS-PAGE, and the transmission electron microscopy (TEM) were performed to characterize the RBC membrane coating.

### *In vitro* release of LY364947

The LPM solution (5 mg mL^-1^) was dialyzed against 50 mL of PBS (pH 7.4) or (pH 5.5). Then, 0.5 mL dialysis solution was collected at selected time points (0 h, 0.5 h, 1 h, 1.5 h, 3 h, 6 h, 12 h and 24 h). Each sample solution was determined by high performance liquid chromatography-ultraviolet spectrometry (HPLC-UV) to calculate the LY364947 release.

### Determination of ^1^O_2_

^1^O_2_ was detected using the singlet oxygen sensor green (SOSG) assay. Briefly, 10 μL of SOSG working solution (5 mM) was added into 500 μL of LPM aqueous suspension (50 μg mL^-1^). Subsequently, the mixed solution was irradiated with ultrasound (US), and the fluorescence intensity (λ_Ex_ = 504 nm/λ_Em_ = 550 nm) of the mixed solution was recorded.

### Cell experiments

NIH-3T3 cells were cultured in DMEM medium containing 1% penicillin-streptomycin and 10% heat-inactivated FBS with 21% O_2_ and 5% CO_2_. 4T1 cells were cultured in RPMI-1640 medium containing 1% penicillin-streptomycin and 10% heat-inactivated FBS with 21% O_2_ and 5% CO_2_, and were chosen as model in all the following cell experiments to evaluate the function and property of LPM. Concentrations of DAC and LPM were 1.25 μM and 50 μg mL^-1^, respectively. Parameters of US irradiation are 0.5 W cm^-2^, 50% duty ratio, 1 MHz. The US exposure time is 5 min.

### Intracellular ROS generation

The 4T1 cells were planted into a 96-well plate and cultured for 12 h. Then the cells were divided into four groups, including PBS-, US-, LPM-treated groups (LPM: 50 μg mL^-1^, LY364947 concentrations: 4.3 μg mL^-1^), and LPM-treated group with US irradiation. After incubation for 6 h, each well was washed with RPMI-1640 culture media to remove free LPM. The 2',7'-dichlorofluorescein diacetate (DCFH-DA) probe (DCFH-DA: 10 μM) was then added to each well and incubated for another 30 min. Next, cells were irradiated by US for 5 min. Finally, the fluorescence images of cells were observed by the inverted fluorescence microscope after cells were washed with PBS.

### MTT assay

The 4T1 cells were planted into a 96-well plate and cultured for 12 h. Then the cells were treated for 24 h with: (G1) PBS, (G2) DAC (DAC: 1.25 μM), (G3) DAC + LPM (LPM: 50 μg mL^-1^, LY364947 concentrations: 4.3 μg mL^-1^), (G4) LPM + US, (G5) DAC + LPM + US in the fresh media. Cells were cultured for another 12 h followed by US irradiation for 5 min. Then, cell viability was measured by MTT assay.

### *In vitro* Annexin V-FITC/PI assay

To evaluate the cell death modalities, the Annexin V-FITC/PI assay was carried out. The 4T1 cells were seeded into a 96-well plate and cultured for 12 h. Then the cells were treated for 24 h with: (G1) PBS, (G2) DAC (DAC: 1.25 μM), (G3) DAC + LPM (LPM: 50 μg mL^-1^, LY364947 concentrations: 4.3 μg mL^-1^), (G4) LPM + US, (G5) DAC + LPM + US in the fresh media. Followed by US irradiation for 5 min, the cells were cultured for another 12 h, and then stained with Annexin V-FITC apoptosis detection kit (20 min). Finally, the cells were analyzed by flow cytometry.

### Living/dead cell double staining assay

To evaluate the cytotoxicity of different treatment groups, the Calcein-AM/PI staining kit was applied for living/dead cell staining. 4T1 cells were seeded into 96-wells plate, and cultured for 12 h. Then the cells were treated as described above for 24 h. Followed by US irradiation for 5 min, the cells were cultured for another 12 h. At last, the cells were stained with Calcein-AM and PI for 10 min, washed with PBS, and observed by inverted fluorescence microscope.

### Morphological study of pyroptosis

4T1 cells were seeded into a 12-well plate and treated as described above. After US irradiation for 5 min, the cells were cultured for another 12 h. Then, the cell morphology was observed using a bright-field optical microscopy. The pyroptotic cell exhibits swelling and bubble-like morphologies.

### Western blot assay

Cells treated in different groups above and lysed in 1 × Laemmli buffer and denatured at 95 °C for 5 min. Cell lysates were separated by 10% SDS-PAGE and transferred onto polyvinylidene fluoride membranes. Blots were incubated with anti-full length GSDME (E2X7E), anti-*N*-terminus fragment of GSDME (EPR19859), anti-cleaved caspases-3 (Asp175) (5A1E) (all 1:1000 dilution), and anti-β-actin (1:5000 dilution) as control. As a secondary antibody, anti-rabbit-IgG-HRP (1:5000 dilution) was used. ECL signal was recorded on the ChemiDoc XRS Biorad Imager.

### LDH and ATP release assay

The culture medium in different groups was harvested, and the supernatant was collected after centrifugation (400 g for 5 min). The amounts of lactate dehydrogenase (LDH) and ATP released were evaluated with LDH Release Assay Kit and ATP Assay Kit (Beyondtime) by the manufacturer's instructions, respectively.

### *In vivo* experiments

In order to establish the mouse tumor model, 4-week-old BALB/c female mice were subcutaneously injected with 5 × 10^5^ 4T1 cells on right hind limb. The tumor-bearing mice were used when the tumor volume exceeded 100 mm^3^. In the following *in vivo* experiments: DAC, 0.35 mg kg^-1^ for 3 days; PM or LPM, 5 mg kg^-1^ for 3 days; US irradiated with 0.5 W cm^-2^, 50% duty ratio, 1 MHz for 10 min.

### *In vivo* tumor targeting

4T1 tumor-bearing mice were randomly divided into 2 groups (n = 5) and intravenously injected with lipophilic dye 1,1-dioctadecyl-3,3,3,3-tetramethylindotricarbocyanine iodid (DiR)-labled LP@liposome (DiR-LPL) (DiR-LPL: 5 mg kg^-1^, containing LY364947 : 0.43 mg kg^-1^) or DiR-labled LP@RBC membrane (DiR-LPM) nanoparticles (DiR-LPM: 5 mg kg^-1^, containing LY364947: 0.43 mg kg^-1^), respectively. After narcotizing with isoflurane, fluorescence images of mice at different time points was captured by live animal imaging system (IVIS). The mice were sacrificed post monitoring for 48 h, the major organs (heart, liver, spleen, lung and kidney) and tumor tissues were collected for *ex vivo* imaging.

### *In vivo* therapeutic experiments

4T1 tumor-bearing mice were randomly divided into seven groups (n = 5), and treated with: (G1) PBS, (G2) DAC (DAC: 0.35 mg kg^-1^), (G3) PM (PM: 5 mg kg^-1^), (G4) LPM (LPM: 5 mg kg^-1^, containing LY364947: 0.43 mg kg^-1^), (G5) PM + US, (G6) DAC + PM + US and (G7) DAC + LPM + US, respectively. Measurements of tumors were performed every 2 days using calipers. Tumor inhibition rate is defined as the ratio of tumor weight in the experimental group to that in the blank control group at the end of the experiment.

### Anti-tumor immune response analysis

For the analysis of pyroptotic-induced immune response, tumors from mice were collected 5 day after treatment. The tumors and spleens were dissociated in RPMI containing 80 μg mL^-1^ Liberase TL and 250 U mL^-1^ DNase I to obtain single cells. Each sample was added with relevant fluorescent conjugated antibodies and incubated at 4 °C for 30 min. Relative immune cells were analysis by fluorescence-activated cell sorting (FACS).

### *In vivo* tumor re-challenge experiments

To demonstrate generation of immune memory, *in vivo* therapeutic experiment was re-conducted. When tumors reached an average volume of 100 mm^3^, the tumor-bearing mice were randomized into 4 groups (n =5) and treated with (G1) PBS, (G2) PM + US (PM: 5 mg kg^-1^), (G3) DAC + PM + US (DAC: 0.35 mg kg^-1^), (G4) DAC + LPM + US (LPM: 5 mg kg^-1^, containing LY364947: 0.43 mg kg^-1^), respectively. The tumors were removed after two-week treatment. Then, the tumor free mice were re-challenged with 2.5 × 10^5^ of 4T1 cells at day 60 after the first tumor removed. Measurements of tumors were performed every 2 days. Three weeks post tumor re-challenge, spleens of mice were collected and digested into single splenocyte for further FACS analysis of memory lymphocytes.

### Statistical analysis

Statistics were performed on SPSS17.0 statistical analysis software. All the error bars indicated mean ± standard deviation. Statistical analysis was performed using Student's t test (two-tailed). Statistical significance was established as indicated * *P* < 0.05; ** *P* < 0.01 and *** *P* < 0.001.

## Results and Discussion

### Characterization of LPM

**Figure 1:**
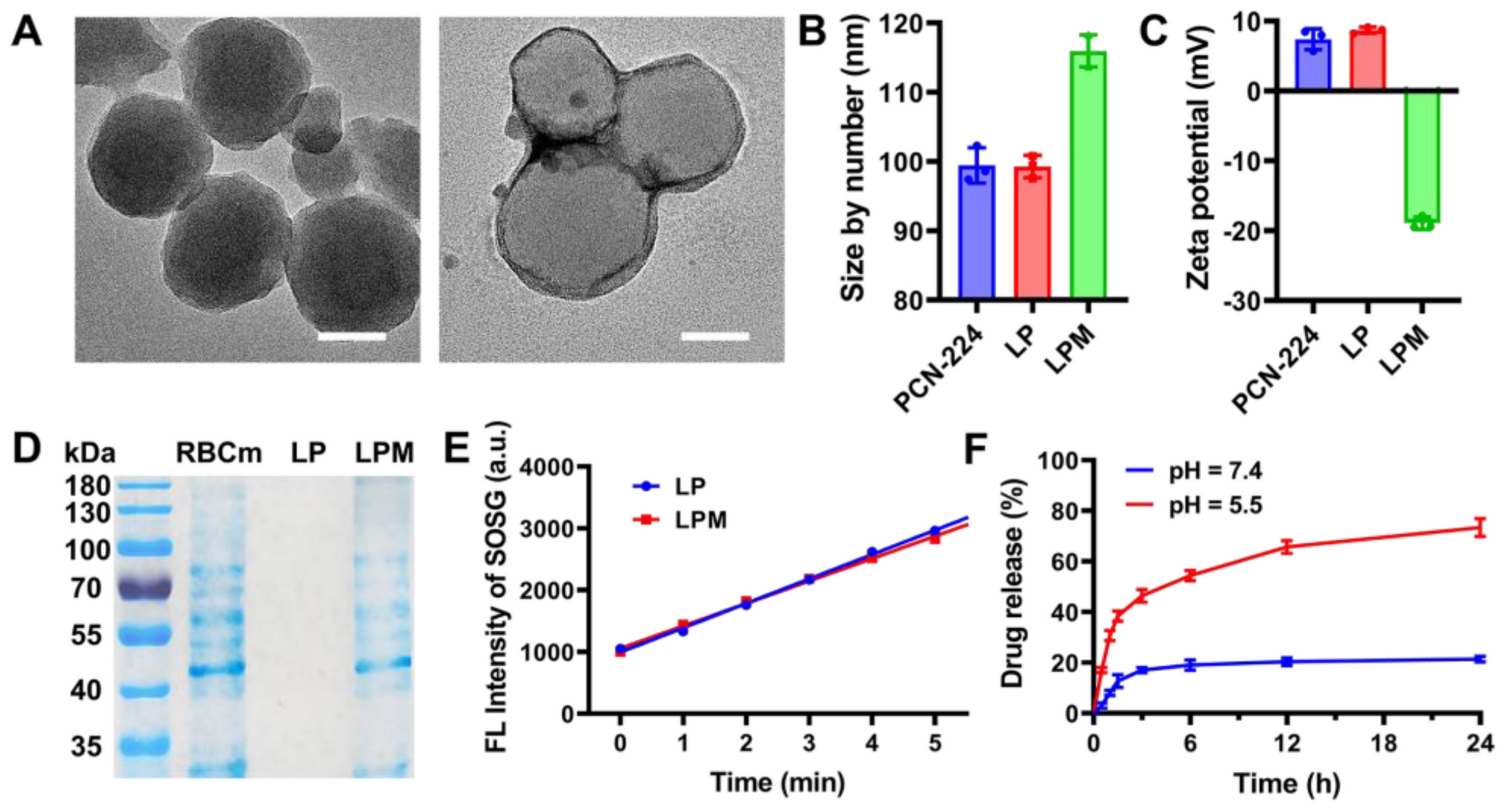
Characterization of LPM. (A) TEM images of PCN-224 (left) and LPM (right). scale bars: 50 nm. (B) Hydrodynamic diameter and (C) Zeta potential of PCN-224, LP and LPM. (D) Protein analysis of RBC membrane, PCN-224 and LPMby SDS-PAGE. (E) Variation of SOSG intensity as a function of ultrasonic irradiation exposure time (0-5 min) for LP and LPM. (F) *In vitro* release of LY364947 from LPM in PBS at pH 5.5 or pH 7.4.

Zirconium-based porphyrinic framework nanoparticles, PCN-224, were chosen as sonosensitizers owing to their effective ROS generation under US 28 and were synthesized using the hydrothermal method. As shown in the TEM image (Figure 1A), PCN-224 nanoparticles were spherical with a controllable average diameter of approximately 90 nm to ensure an enhanced permeability and retention (EPR) effect 29. To normalize the ECM and infiltrate-excluded tumor immunosuppression microenvironment, PCN-224 nanoparticles further encapsulated the TGFβRI inhibitor, LY364947, with a loading efficiency of 8.6 wt%. To guarantee longer circulation and tumor-targeting ability *in vivo*, LY364947@PCN-224 (LP) nanoparticles were coated with RBC membranes by sonication to obtain camouflaged LY364947@PCN-224@membrane (LPM) nanoparticles. As shown in Figure 1B, LPM had a hydrodynamic diameter of 116.0 ± 2.3 nm, 16.7 nm larger than that of bare LP. Meanwhile, the surface charge of LPM shifted to negative (from 8.7 ± 0.5 mV to -18.9 ± 0.9 mV, Figure 1C). The changes in size and zeta potential may be attributed to RBC membrane coating. Furthermore, SDS-PAGE results indicated that the composition of the membrane proteins and their biological activities were well retained (Figure 1D), confirming that LPM was successfully fabricated. No significant changes in sonosensitivity, measured using the fluorescence of singlet oxygen sensor green (SOSG), were observed after RBC membrane coating (Figure 1E and Figure S1), indicating that RBC membrane coating barely affected the sonosensitivity of LP.

Biostability of LPM was also evaluated by measuring its hydrodynamic size. The hydrodynamic diameter of the LPM showed negligible change after incubation in PBS or serum for 7 d (Figure S2), demonstrating good stability of LPM. To ensure the biological safety of LPM, MTT assay and hemolysis test were carried out. The result indicated that LPM with a concentration of 160 mg L^-1^ was almost non-toxic in NIH-3T3 cells (Figure S3A) and LPM with a concentration of 160 mg L^-1^ also did not show hemolysis (Figure S3B). In addition, the encapsulated LY364947 molecules were released from the LPM skeleton upon exposure to acidic conditions. This release behavior could be attributed to the loss of coordinating capability between H_2_TCPP and Zr clusters caused by protonation of H_2_TCPP in acidic condition. The release rate of LY364947 was 76.6% within 24 h at pH 5.5 (simulating lysosomes) compared to 19.2% at pH 7.4, suggesting that LPM could serve as a good drug carrier to achieve controllable release of LY364947 molecules (Figure 1F).

### Pyroptosis triggering *in vitro* by SDT

Recently, it has been reported that GSDME, a member of the gasdermin family, can be specifically cleaved by caspase-3, leading to pyroptosis in both human and murine cells by forming pores on the cell membrane. However, due to methylation of DNA promoter, GSDME expression in most tumor cells is silenced. To better represent pyroptosis, DAC was selected to demethylate the deafness autosomal dominant 5 (DFNA5) gene and subsequently upregulate GSDME (encoded by DFNA5) in 4T1 tumor cells 30, 31.

**Figure 2:**
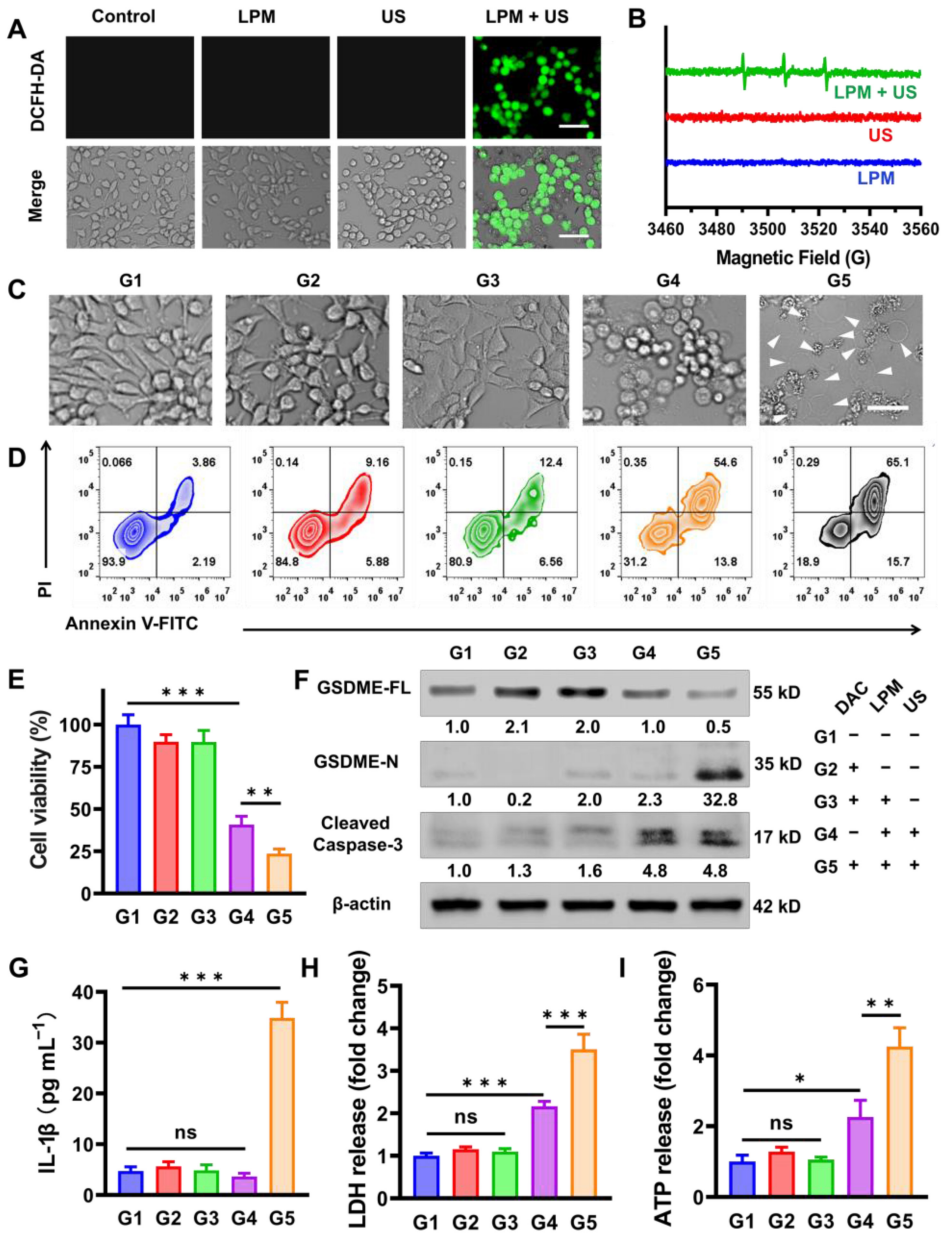
Pyroptosis triggering *in vitro* by SDT. (A) Fluorescence images of cellular ROS production induced by SDT detected by the DCFH-DA probe. Scale bar: 100 μm. (B) ESR spectra demonstrating the ^1^O_2_ generation of LPM with or without US irradiation (1.0 MHz, 0.5 W cm^-2^, 5 min). (C) Representative photographs of 4T1 cells after different treatments. The differently treated groups are as follows: (G1) PBS, (G2) DAC (DAC: 1.25 μM), (G3) DAC + LPM (LPM: 50 μg mL^-1^, LY364947 concentrations: 4.3 μg mL^-1^), (G4) LPM under US irradiation, (G5) DAC + LPM under US irradiation. Scale bar: 50 μm. (D) Flow cytometry analysis of Annexin V-FITC and PI co-stained 4T1 cells after different treatments. (E) The cytotoxicity assessment on 4T1 cells after different treatments. (F) Western blot analysis of GSDME-FL, GSDME-N and cleaved caspase-3 expression in 4T1 cells after different treatments. The release of IL-1β (G), LDH (H) and ATP (I) from 4T1 cells after different treatments. Data are presented as the mean ± SD. Statistical significance was calculated via unpaired t-test. (ns, nonsignificant; *P < 0.05; **P < 0.01; ***P < 0.001).

Before *in vitro* pyroptotic therapy, the SDT capability of LPM was investigated in 4T1 cells by detecting singlet oxygen with DCFH-DA probe and electron spin resonance (ESR) spectra. Under conditions without US irradiation, LPM and DAC exhibited negligible cytotoxicity at concentrations of up to 100 mg L^-1^ and 5.0 μM, respectively (Figure S4A and Figure S4B). Therefore, a concentration of 50 mg L^-1^ LPM and 1.25 μM DAC was chosen for subsequent intracellular experiments. Safe US intensity was used to be 0.5 W cm^-2^ for 5 min (Figure S4C). The merging of SDT resulted in a considerable increase in ROS generation, reflected by remarkable intracellular fluorescence of DCF in the LPM with US group (Figure 2A). The singlet oxygen generated was determined using ESR (Figure 2B). The LPM exhibited a high SDT efficiency *in vitro*.

To verify whether LPM could trigger pyroptosis, cell morphology was observed using bright-field optical microscopy (Figure 2C). Cells showed typical apoptotic morphology with round and shrunken cells in the LPM with US group, indicating SDT-induced apoptosis. No obvious changes in cell morphology were observed in the DAC or LPM groups compared to the blank control (PBS) group. In contrast, a population of cells showed characteristic pyroptotic bubble-like morphology with 1.25 μM DAC plus LPM under US irradiation. These results suggested the potential of LPM to induce pyroptosis upon US irradiation in cells with high GSDME expression. The cytotoxicity measured using MTT assay showed that cells treated with LPM and US exhibited nearly 60% cell death, while those in the DAC plus LPM with US group showed 76% cell death (Figure 2E). Calcein-AM/PI staining and Annexin V-FITC/PI staining FACS assays confirmed efficient cell death induced by LPM (Figure S5 and Figure 2D).

To further validate the pyroptosis process during treatment, Western blot analysis was performed. As shown in Figure 2F, intracellular GSDME expression was upregulated by 1.8-fold upon the treatment with DAC for 24 h (G2 and G3 group). Subsequently, both cleaved caspase-3 and the N-terminus fragment of GSDME (GSDME-N) significantly increased in the DAC plus LPM group upon US irradiation (G5 group), indicating a pyroptotic process. Meanwhile, an approximately 75% decrease in full-length GSDME (GSDME-FL) was observed. In contrast, in the absence of US irradiation or DAC pretreatment group, none of the groups showed variations in the GSDME-N levels. Among them, LPM plus US without DAC pretreatment group (G4 group) only showed an increase in the expression of cleaved caspase-3, indicating that apoptosis occurred. These results suggest that SDT successfully triggered pyroptotic processes via the caspase-3/GSDME pathway in DAC pretreated group, indicative of the co-regulation of DAC pretreatment and SDT therapy on pyroptosis.

Since GSDME-N was expected to trigger membrane pore formation and release of pro-inflammatory cytokines, the interleukin 1 beta (IL-1β) and interleukin 18 (IL-18) content in each group was measured using the enzyme linked immunosorbent assay (ELISA). Compared to that in the PBS group, as shown in Figure 2G and Figure S6, IL-1β and IL-18 leakages were sharply elevated by approximately 7-fold in the DAC plus LPM with US group (G5 group), as expected under conditions of GSDME-dependent pore formation. Additionally, LDH and ATP, which are markers of pyroptosis 32, 33, were observed to leak progressively into the extracellular fluid compartment 12 h after treatment (5 μM LPM and 0.5 mW cm^-2^ US irradiation for 5 min), as shown in Figure 2H-I, and were expected to enhance the subsequent anti-tumor innate immune activation process.

### Normalization of tumor physical scaffold by collagen removing

**Figure 3:**
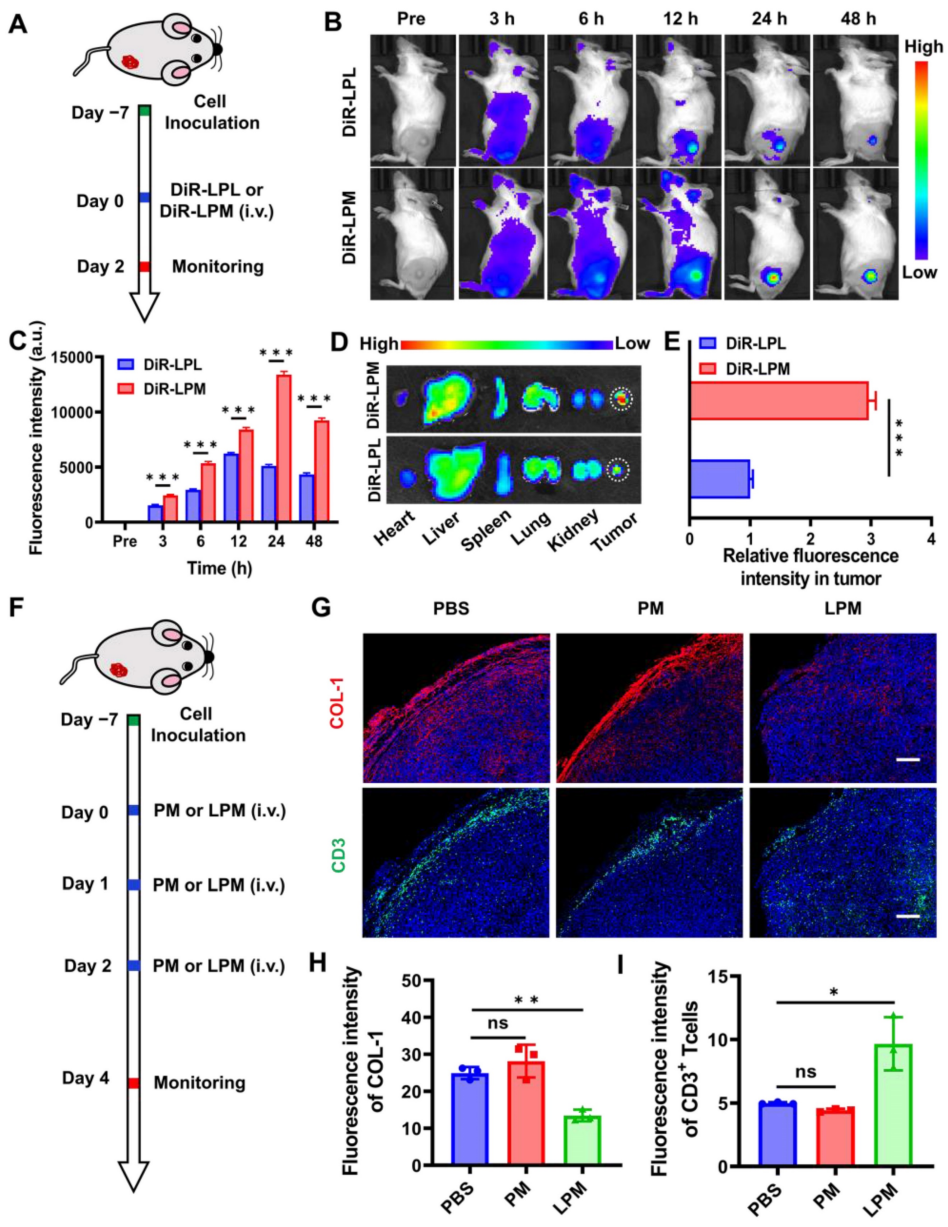
Normalization of tumor physical scaffold by collagen removing. (A) Schematic showing the tumor targeting of nanoparticles in 4T1 tumor-bearing mouse model. (B) *In vivo* fluorescence imaging for 4T1 tumor-bearing mice at different time points post-injection with DiR-LPL (DiR-LPL: 5 mg kg^-1^, containing LY364947: 0.43 mg kg^-1^) and DiR-LPM (DiR-LPM: 5 mg kg^-1^, containing LY364947: 0.43 mg kg^-1^). (C) Average fluorescence intensity of tumor tissue at different time points. (D) Ex vivo fluorescence images of the major organs (heart, liver, spleen, lungs, and kidneys) and tumors at 24 h post-injection with DiR-LPL and DiR-LPM. (E) Average fluorescence intensity of tumor tissue at 24 h post-injection with DiR-LPL and DiR-LPM. (F) Schematic showing the collagen removing by LPM in 4T1 tumor-bearing mouse model (PM:5 mg kg^-1^; LPM: 5 mg kg^-1^, containing LY364947: 0.43 mg kg^-1^). Representative images of immunofluorescent staining (G) and quantification of collagen I (H) and CD3^+^T cells (I) in 4T1 tumors. Blue, cell nuclei staining; red, collagen I staining; green, CD3^+^T cells staining. Scale bar: 200 μm. Data are presented as the mean ± SD. Statistical significance was calculated via unpaired t-test. (ns, nonsignificant; *P < 0.05; **P < 0.01; ***P < 0.001).

The *in vivo* biodistribution of LPM was first investigated using an IVIS. LPM was labeled with the lipophilic near-infrared (NIR) fluorescent dye DiR, which was immobilized in the hydrophobic domain of the membrane bilayer. 4T1 tumor-bearing mice were administrated with DiR-LPL or DiR-LPM and imaged at 3, 6, 12, 24, and 48 h post-injection (Figure 3A). NIR fluorescent signals were distinctly observed at the tumor site 6 h after *i.v.* injection of DiR-LPM (100 µL, 1 mg mL^-1^) and reached a maximum at approximately 24 h (Figure 3B). Despite exhibiting a similar organ distribution relative to DiR-LPL, the high fluorescence intensity of the tumor site in the LPM group was observed to confirm the ability of the mononuclear phagocyte system to escape conferred by surface-coated RBC membrane camouflage (Figure 3C). An 8.62% total enrichment of LPM (Figure S7) and a 2.96-fold increase over the LPL treatment in accumulation (Figure 3D-E) were detected, indicating that LPM could potentially be used for subsequent tumor-targeted drug delivery and pyroptotic therapy.

Having confirmed the accumulation of LPM in the tumors, the normalization of ECM was explored in subcutaneous 4T1 tumor mouse models (Figure 3F). Collagen constitutes the physical scaffold of the tumor microenvironment and leads to dense ECM, which is one of the major obstacles to tumor immunotherapy 34, 35. Thus, depletion of collagen should help normalize infiltrated-excluded tumor immunosuppression microenvironment and further promote the deep infiltration of lymphocytes. To investigate and demonstrate this phenomenon, PCN@RBC membranes without LY364947 loading (PM) and LPM were injected on day 0, 1, and 2, and the collected tumor tissues were analyzed using immunofluorescence staining. Representative images of collagen at the edge of the 4T1 tumor showed a significant decrease in collagen in the LPM group compared to that in the PM group (Figure 3G). This finding was accompanied by a 48% collagen depletion and a 1.9-fold increase in tumor-infiltrating lymphocytes with a more uniform distribution (Figure 3H-I), indicating its potential in amplifying the suite of concomitant immune responses elicited by pyroptosis subsequently.

### Anti-tumor effect of SDT-triggered pyroptotic therapy *in vivo*

**Figure 4:**
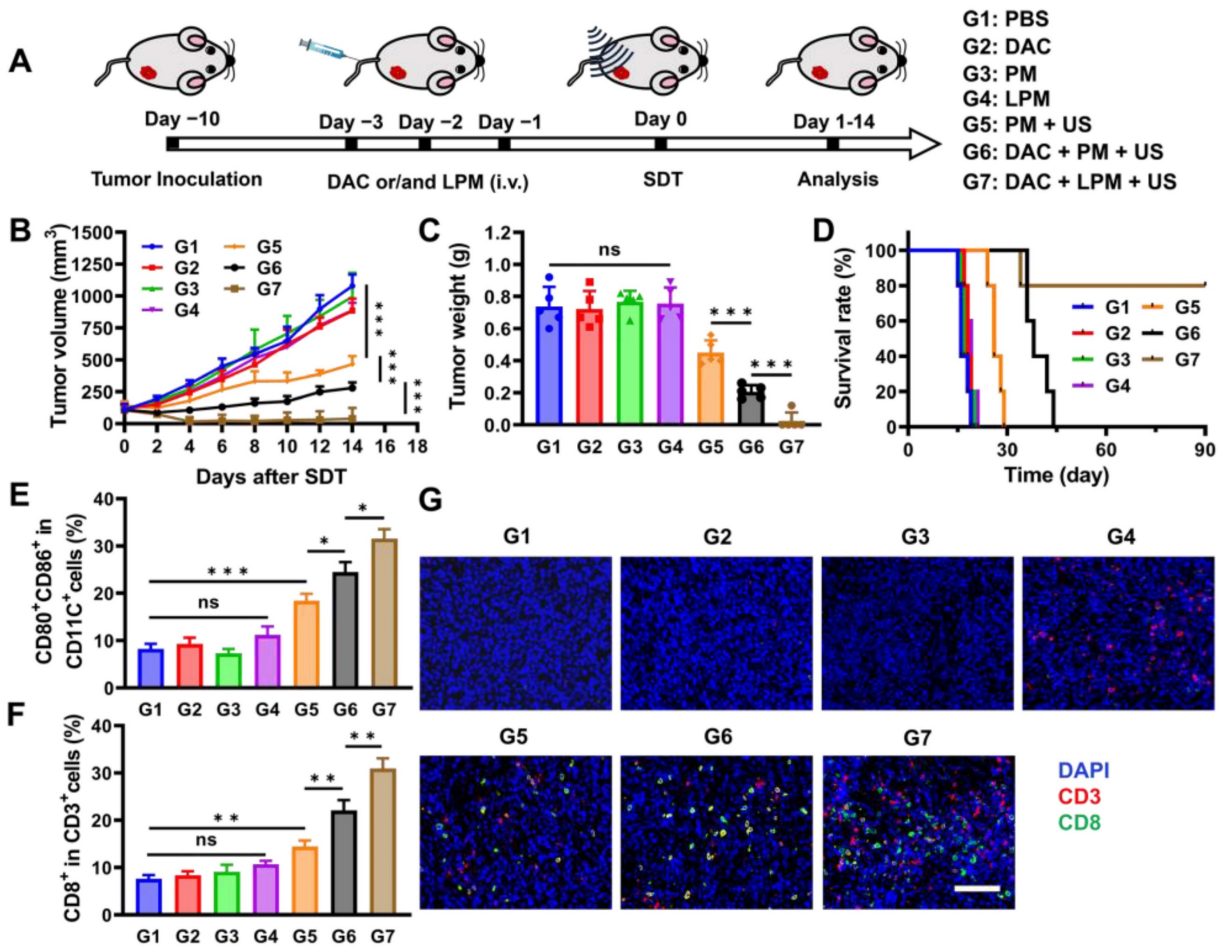
Anti-tumor effect of SDT-triggered pyroptotic therapy *in vivo*. (A) Schematic illustration of treatment schedules of 4T1 tumor-bearing mouse model. (B) Tumor growth kinetics by different treatments (n = 5) as follows: (G1) PBS, (G2) DAC (DAC: 0.35 mg kg^-1^), (G3) PM (PM: 5 mg kg^-1^), (G4) LPM (LPM: 5 mg kg^-1^, containing LY364947: 0.43 mg kg^-1^), (G5) PM under US irradiation, (G6) DAC + PM under US irradiation and (G7) DAC + LPM under US irradiation. (C) Tumor weight of mice after different treatments at day 14. (D) Survival curves of different treatment groups within 90 days. Absolute quantification of CD80^+^CD86^+^ cells gating on CD11c^+^ cells in TDLNs (E) and CD8^+^ T cells gating on CD3^+^ T cells in tumors (F) after different treatments. (G) Immunofluorescence staining of tumor tissues for CD3 (red) and CD8 (green). Scale bar: 200 μm. Data are presented as the mean ± SD. Statistical significance was calculated via unpaired t-test. (ns, nonsignificant; *P < 0.05; **P < 0.01; ***P < 0.001).

In light of the immunosuppressive microenvironment removal and efficient SDT-induced pyroptosis, LPM was examined for its *in vivo* anti-tumor activity. Mice bearing 100 mm^3^ 4T1-tumors were divided into seven groups: (G1) blank control (PBS), (G2) DAC (0.25 mg kg^-1^), (G3) PM (5 mg kg^-1^), (G4) LPM (5 mg kg^-1^), (G5) PM plus US irradiation (0.5 mW cm^-2^, 10 min), (G6) PM plus US irradiation after DAC pretreatment, and (G7) LPM plus US irradiation after DAC pretreatment. After *i.v.* injection once a day for 3 d, mice were subjected to US irradiation on tumor areas and monitored for another 14 d (Figure 4A). Compared to the PBS group, no significant tumor inhibition was detected in the DAC-, PM-, or LPM-treated groups (Figure 4B). However, PM with US group (equivalent to typical SDT) showed significant tumor inhibition, with an average tumor weight decrease of 41.8%. The DAC plus PM with US group, which is supposed to mimic pyroptotic therapy, showed remarkable suppression of tumor growth, with tumor weight decrease of 69.7%. Notably, the DAC plus LPM with US group showed almost complete tumor elimination (Figure 4C). Furthermore, 80% of the mice in DAC plus LPM with US group showed good tumor-free survival at the end of the study (Figure 4D). Together, these results confirmed the potent anti-tumor efficacy of LPM-induced pyroptotic therapy in combination with US.

To verify the occurrence of pyroptosis at animal level, Western blot and ELISA assay were carried out. As shown in Figure S8A, the expression of GSDME-N increased only in G6 and G7 groups, indicating that pyroptosis occurred in G6 and G7 groups. The content of IL-1β (Figure S8B) and IL-18 (Figure S8C) in tumor homogenate also confirmed this result. Previous studies have shown that pyroptosis can induce anti-tumor immune responses 36, 37. To investigate the *in vivo* immune effect, cells from tumor tissues and tumor-draining lymph node (TDLN) tissues were collected to detect the proportion of immune cells using flow cytometry. As shown in Figure 4E and Figure S9, the proportion of mature CD11c^+^CD80^+^CD86^+^ dendritic cells (DC) after DAC plus PM treatment (G6 group) with US increased (3.4-fold) more than that of PM treatment only (G3 group). This result indicates that tumor cell pyroptosis could promote DC maturation, benefiting from pyroptosis-induced antigen booming 38. After DC maturation, antigens were presented to T cells, initiating adaptive immune response 39. The proportion of CD8^+^ tumor-infiltrating T cells increased in the DAC plus PM with US group (G6 group). Both DC maturation and CD8+ T cell infiltration indicated that an adaptive immune response was initiated by pyroptotic therapy. Notably, the DAC plus LPM with US group (G7 group) exhibited further promotion of DC maturation, which might benefit from ECM normalization-enhanced antigen presentation 40. Correspondingly, as shown in Figure 4F and Figure S10, a significant (1.3-fold) upregulation of CD8+ tumor-infiltrating lymphocytes was observed in the DAC plus LPM with US group (G7 group) compared to the DAC plus PM with US group (G6 group), suggesting that collagen depletion enhanced the adaptive immune response induced by pyroptosis. This result was supported by the findings of immunofluorescence staining (Figure 4G).

Moreover, there were no obvious changes in body weight or body temperature during the 14 days post-treatment and the blood routine index was within the reference value (Figure S11), indicating the safety of LPM-mediated pyroptotic therapy. Hematoxylin and Eosin (H&E) staining results of main organs in different treatment groups further confirmed the above conclusions (Figure S12).

### Long-term anti-tumor memory immune response

**Figure 5:**
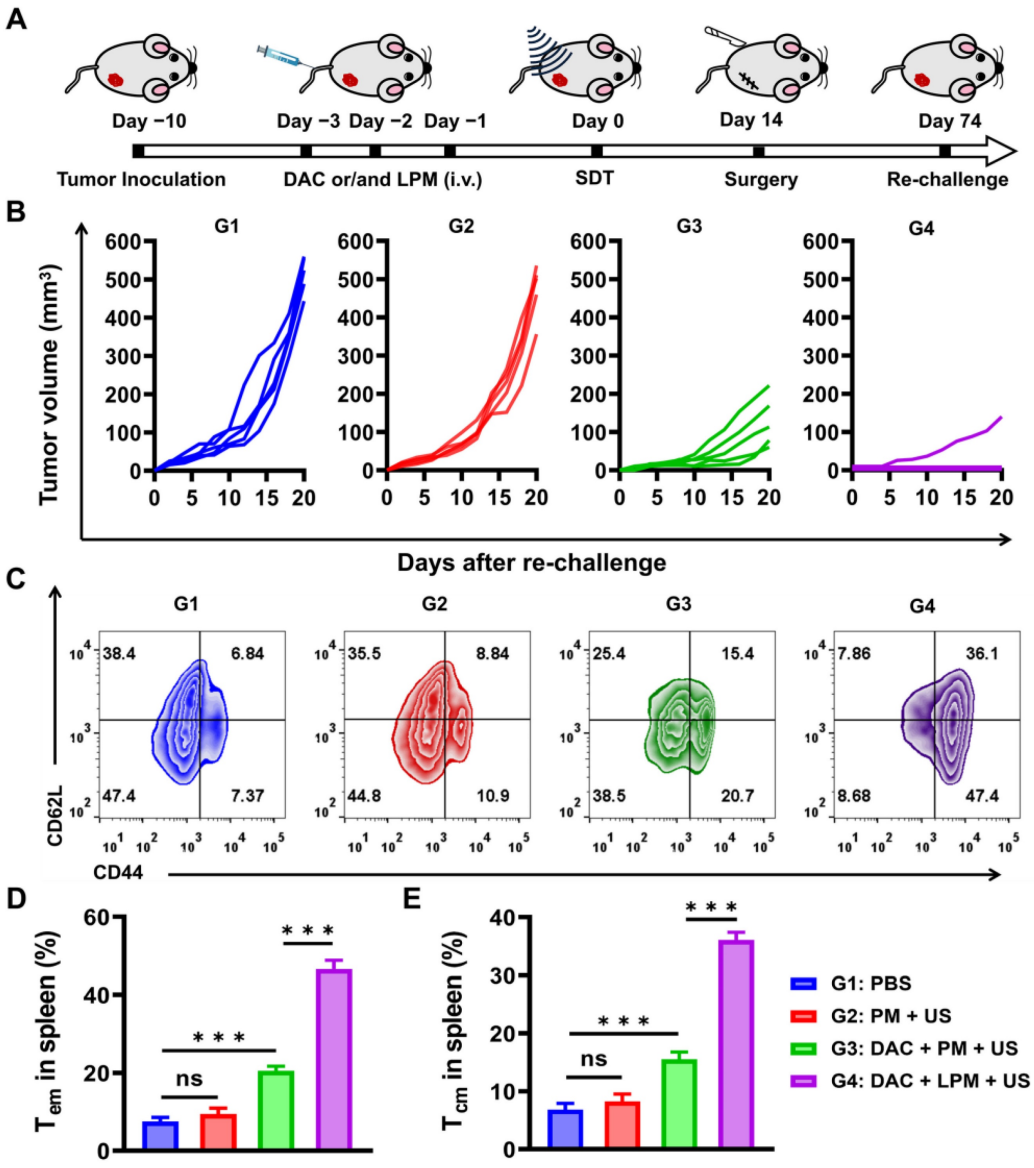
Long-term anti-tumor memory immune response. (A) Schematic illustration of tumor re-challenge assay schedules of 4T1 tumor bearing mouse model. The different treatments are as follows: (G1) PBS, (G2) PM under US irradiation (PM: 5 mg kg^-1^) (G3) DAC + PM under US irradiation (DAC: 0.35 mg kg^-1^; PM:5 mg kg^-1^) and (G4) DAC + LPM under US irradiation (DAC: 0.35 mg kg^-1^; LPM:5 mg kg^-1^, containing LY364947: 0.43 mg kg^-1^). (B) Tumor growth kinetics of tumor re-challenge assay. (C) Representative flow cytometric analysis of memory T cells within the spleen after tumor re-challenge assay. Absolute quantification of CD44^+^CD62L^-^ cells gating on CD3^+^CD4^+^ cells (D) and CD44^+^CD62L^+^ cells gating on CD3^+^CD4^+^ cells (E). Data are presented as the mean ± SD. Statistical significance was calculated via unpaired t-test. (ns, nonsignificant; *P < 0.05; ***P < 0.001).

The release of pro-inflammatory factors and whole-tumor antigens from the pyroptotic cells has been reported to induce innate immune activation 41. Therefore, we hypothesized that sonodynamic-triggered pyroptotic therapy might lead to an adaptive immune response with the establishment of immunological memory. To investigate the effect of pyroptotic therapy on memory T-cell response *in vivo*, we used a tumor re-challenge model. The 4T1 subcutaneous tumor-bearing mice were treated intravenously with(G1) PBS, (G2) PM, (G3) DAC plus PM, or (G4) DAC plus LPM once a day for 3 d. Subsequently, subcutaneous tumors were surgically resected 14 d after US irradiation, and mice were re-challenged on the contralateral flank 60 d later to evaluate the ability of LPM-induced immunological memory to protect against recurrence (Figure 5A). As shown in Figure 5B, for the G3 group, the growth of re-challenge tumors were strongly inhibited, and the proliferation of memory T cells was also increased. This result indicated that pyroptosis can establish a significant anti-tumor immune memory. Furthermore, it was found that 80% of mice in G4 group were completely protected from tumor re-challenge without tumor growth within 3 weeks, indicative of a T-cell memory response. Such a memory response is strongly reflected by a 5.4-fold proliferation of CD62L^+^CD44^+^CD4^+^ central memory T cells (Tcm) and 6.4-fold proliferation of CD62L^-^CD44^+^CD4^+^ effector memory T cells (Tem) in the spleen (Figure 5C-E). These results confirmed the establishment of anti-tumor immune memory by LPM-mediated pyroptotic therapy 42 and highlighted the strong enhancement of anti-tumor immune memory by ECM removal. In contrast, SDT could induce neither re-challenged tumor inhibition nor significant Tcm generation, despite its appreciable anti-tumor effect in subcutaneous models. This finding further illustrated that the immunogenicity and adaptive immune reaction originated from pyroptosis. Taken together, these results provide the first demonstration that SDT-induced pyroptotic therapy can stimulate anti-tumor immunity with the potential to combat tumor recurrence.

## Conclusions

In summary, we presented a sonodynamic-immunomodulatory strategy, which was not only able to initiate pyroptosis by combination of DAC and SDT, but also amplified pyroptosis-mediated tumor immunotherapy by remodeling of the tumor immune microenvironment. To implement this strategy, we rationally designed a multi-functional nanostimulator LPM, which is composed of PCN-224 skeleton for SDT and encapsulated LY364947 for depletion of collagen from the ECM. Our results revealed that the LPM with high sonosensitivity not only exhibited excellent ROS generation capability for the highly efficient SDT, but also can activate caspase-3/GSDME-dependent pyroptosis pathway in DAC-pretreated 4T1 cells, resulting in antigen booming with strong DC maturation. More importantly, LY364947 encapsulated within LPM substantially impeded formation of ECM by depletion of collagen and facilitated the T-lymphocyte infiltration, which further amplified pyroptosis-mediated tumor immnotherapy. In the 4T1 breast cancer model with low immunogenicity and infiltrated-excluded tumor immune microenvironment, LPM benefited from our sonodynamic-immunomodulatory strategy and achieved nearly complete tumor eradication with the formation of immunological memory and prolonged survival time. This study offers a novel strategy for pyroptosis-mediated tumor immunotherapy, which provides a wider vision of pyroptosis in cancer immunology and deepens our understanding of immune behaviors of sonosensitizer.

## Supplementary Material

Supplementary figures.

## References

[1] Shi J, Zhao Y, Wang K, Shi X, Wang Y, Huang H (2015). Cleavage of GSDMD by inflammatory caspases determines pyroptotic cell death. Nature.

[2] Rao Z, Zhu Y, Yang P, Chen Z, Xia Y, Qiao C (2022). Pyroptosis in inflammatory diseases and cancer. Theranostics.

[3] Zhang Z, Zhang Y, Xia S, Kong Q, Li S, Liu X (2020). Gasdermin E suppresses tumour growth by activating anti-tumour immunity. Nature.

[4] Wang Y, Gao W, Shi X, Ding J, Liu W, He H (2017). Chemotherapy drugs induce pyroptosis through caspase-3 cleavage of a gasdermin. Nature.

[5] Fan JX, Deng RH, Wang H, Liu XH, Wang XN, Qin R (2019). Epigenetics-Based Tumor Cells Pyroptosis for Enhancing the Immunological Effect of Chemotherapeutic Nanocarriers. Nano Lett.

[6] Xiao Y, Zhang T, Ma X, Yang QC, Yang LL, Yang SC (2021). Microenvironment-Responsive Prodrug-Induced Pyroptosis Boosts Cancer Immunotherapy. Adv Sci.

[7] Liang MY, Zhang MJ, Qiu W, Xiao Y, Ye MJ, Xue P (2022). Stepwise Size Shrinkage Cascade-Activated Supramolecular Prodrug Boosts Antitumor Immunity by Eliciting Pyroptosis. Adv Sci.

[8] Wu M, Liu X, Chen H, Duan Y, Liu J, Pan Y (2021). Activation of Pyroptosis by Membrane-Anchoring AIE Photosensitizer Design: New Prospect for Photodynamic Cancer Cell Ablation. Angew Chem Int Ed Engl.

[9] Wang M, Wu M, Liu X, Shao S, Huang J, Liu B (2022). Pyroptosis Remodeling Tumor Microenvironment to Enhance Pancreatic Cancer Immunotherapy Driven by Membrane Anchoring Photosensitizer. Adv Sci.

[10] Yu L, Xu Y, Pu Z, Kang H, Li M, Sessler JL (2022). Photocatalytic Superoxide Radical Generator that Induces Pyroptosis in Cancer Cells. J Am Chem Soc.

[11] Chen B, Yan Y, Yang Y, Cao G, Wang X, Wang Y (2022). A pyroptosis nanotuner for cancer therapy. Nat Nanotechnol.

[12] Wu D, Wang S, Yu G, Chen X (2021). Cell Death Mediated by the Pyroptosis Pathway with the Aid of Nanotechnology: Prospects for Cancer Therapy. Angew Chem Int Ed Engl.

[13] Son S, Kim JH, Wang X, Zhang C, Yoon SA, Shin J (2020). Multifunctional sonosensitizers in sonodynamic cancer therapy. Chem Soc Rev.

[14] Yang H, Tu L, Li J, Bai S, Hu Z, Yin P (2022). Deep and precise lighting-up/combat diseases through sonodynamic agents integrating molecular imaging and therapy modalities. Coordin Chem Rev.

[15] Jiang F, Yang C, Ding B, Liang S, Zhao Y, Cheng Z (2022). Tumor microenvironment-responsive MnSiO3-Pt@BSA-Ce6 nanoplatform for synergistic catalysis-enhanced sonodynamic and chemodynamic cancer therapy. Chinese Chem Lett.

[16] Li D, Yang Y, Li D, Pan J, Chu C, Liu G (2021). Organic Sonosensitizers for Sonodynamic Therapy: From Small Molecules and Nanoparticles toward Clinical Development. Small.

[17] Qian X, Zheng Y, Chen Y (2016). Micro/Nanoparticle-Augmented Sonodynamic Therapy (SDT): Breaking the Depth Shallow of Photoactivation. Adv Mater.

[18] Zhang T, Jia Y, Yu Y, Zhang B, Xu F, Guo H (2022). Targeting the tumor biophysical microenvironment to reduce resistance to immunotherapy. Adv Drug Deliv Rev.

[19] Lecker LSM, Berlato C, Maniati E, Delaine-Smith R, Pearce OMT, Heath O (2021). TGFBI Production by Macrophages Contributes to an Immunosuppressive Microenvironment in Ovarian Cancer. Cancer Res.

[20] Meng Xm, Nikolic-Paterson D (2016). & Lan, H. TGF-β: the master regulator of fibrosis. Nat Rev Nephrol.

[21] Qiao C, Wang X, Liu G, Yang Z, Jia Q, Wang L (2021). Erythrocyte Membrane Camouflaged Metal-Organic Framework Nanodrugs for Remodeled Tumor Microenvironment and Enhanced Tumor Chemotherapy. Adv Funct Mater.

[22] Wang Y, Gao Z, Du X, Chen S, Zhang W, Wang J (2020). Co-inhibition of the TGF-beta pathway and the PD-L1 checkpoint by pH-responsive clustered nanoparticles for pancreatic cancer microenvironment regulation and anti-tumor immunotherapy. Biomater Sci.

[23] Yang Z, Qiao C, Jia Q, Chen Z, Wang X, Liu X (2022). Redox dyshomeostasis modulation of the tumor intracellular environment through a metabolic intervention strategy for enhanced photodynamic therapy. Theranostics.

[24] Bao Y, Chen J, Qiu H, Zhang C, Huang P, Mao Z (2021). Erythrocyte Membrane-Camouflaged PCN-224 Nanocarriers Integrated with Platinum Nanoparticles and Glucose Oxidase for Enhanced Tumor Sonodynamic Therapy and Synergistic Starvation Therapy. ACS Appl Mater Interfaces.

[25] Miao Y, Yang Y, Guo L, Chen M, Zhou X, Zhao Y (2022). Cell Membrane-Camouflaged Nanocarriers with Biomimetic Deformability of Erythrocytes for Ultralong Circulation and Enhanced Cancer Therapy. ACS Nano.

[26] Li SY, Cheng H, Xie BR, Qiu WX, Zeng JY, Li CX (2017). Cancer Cell Membrane Camouflaged Cascade Bioreactor for Cancer Targeted Starvation and Photodynamic Therapy. ACS Nano.

[27] Ben-Akiva E, Meyer RA, Yu H, Smith JT, Pardoll DM, Green JJ (2020). Biomimetic anisotropic polymeric nanoparticles coated with red blood cell membranes for enhanced circulation and toxin removal. Sci Adv.

[28] Wang Z, Liu B, Sun Q, Feng L, He F, Yang P (2021). Upconverted Metal-Organic Framework Janus Architecture for Near-Infrared and Ultrasound Co-Enhanced High Performance Tumor Therapy. ACS Nano.

[29] Tan J, Li H, Hu X, Abdullah R, Xie S, Zhang L (2019). Size-Tunable Assemblies Based on Ferrocene-Containing DNA Polymers for Spatially Uniform Penetration. Chem.

[30] Zhao P, Wang M, Chen M, Chen Z, Peng X, Zhou F (2020). Programming cell pyroptosis with biomimetic nanoparticles for solid tumor immunotherapy. Biomaterials.

[31] Xie B, Liu T, Chen S, Zhang Y, He D, Shao Q (2021). Combination of DNA demethylation and chemotherapy to trigger cell pyroptosis for inhalation treatment of lung cancer. Nanoscale.

[32] Liu Y, Lu Y, Ning B, Su X, Yang B, Dong H (2022). Intravenous Delivery of Living Listeria monocytogenes Elicits Gasdmermin-Dependent Tumor Pyroptosis and Motivates Anti-Tumor Immune Response. ACS Nano.

[33] Wang H, Rong X, Zhao G, Zhou Y, Xiao Y, Ma D (2022). The microbial metabolite trimethylamine N-oxide promotes antitumor immunity in triple-negative breast cancer. Cell Metab.

[34] Chung SW, Xie Y, Suk JS (2021). Overcoming physical stromal barriers to cancer immunotherapy. Drug Deliv Transl Res.

[35] Hu J, Yuan X, Wang F, Gao H, Liu X, Zhang W (2021). The progress and perspective of strategies to improve tumor penetration of nanomedicines. Chinese Chem Lett.

[36] Wang Q, Wang Y, Ding J, Wang C, Zhou X, Gao W (2020). A bioorthogonal system reveals antitumour immune function of pyroptosis. Nature.

[37] Zhang S, Zhang Y, Feng Y, Wu J, Hu Y, Lin L (2022). Biomineralized Two-Enzyme Nanoparticles Regulated Tumor Glycometabolism Inducing Tumor Cell Pyroptosis and Robust Anti-Tumor Immunotherapy. Adv. Mater.

[38] Zhang Z, Zhang Y, Lieberman J (2021). Lighting a Fire: Can We Harness Pyroptosis to Ignite Antitumor Immunity?. Cancer Immunol Res.

[39] Chen DS, Mellman I (2013). Oncology meets immunology: the cancer-immunity cycle. Immunity.

[40] Grout JA, Sirven P, Leader AM, Maskey S, Hector E, Puisieux I (2022). Spatial Positioning and Matrix Programs of Cancer-Associated Fibroblasts Promote T-cell Exclusion in Human Lung Tumors. Cancer Discov.

[41] Zhou Z, He H, Wang K, Shi X, Wang Y, Su Y (2020). Granzyme A from cytotoxic lymphocytes cleaves GSDMB to trigger pyroptosis in target cells. Science.

[42] Badrinath S, Dellacherie MO, Li A, Zheng S, Zhang X, Sobral M (2022). A vaccine targeting resistant tumours by dual T cell plus NK cell attack. Nature.

