# Supplementary material for: Sonodynamic-immunomodulatory nanostimulators activate pyroptosis and remodel tumor microenvironment for enhanced tumor immunotherapy
Source: Theranostics. 2023 Mar 5;13(5):1571–83. doi: 10.7150/thno.79945 (PMC10086211; doi:10.7150/thno.79945)
Supplement: Supplementary file 1 — Supplementary figures. [file thnov13p1571s1.pdf]

Supporting Information

Sonodynamic-immunomodulatory nanostimulators activate pyroptosis and remodel tumor microenvironment for enhanced tumor immunotherapy

Zhuang Chen[1], Weijing Liu[1], Zuo Yang[1✉], Yi Luo[1], Chaoqiang Qiao[1], Anna Xie[1], Qian Jia[1], Peng Yang[1✉], Zhongliang Wang[1,2✉], Ruili Zhang[1,2✉]

1. Lab of Molecular Imaging and Translational Medicine (MITM), Engineering Research Center of Molecular and Neuro Imaging, Ministry of Education, School of Life Science and Technology, Xidian University & International Joint Research Center for Advanced Medical Imaging and Intelligent Diagnosis and Treatment, Xi'an, Shaanxi, 710126, P. R. China
2. Academy of Advanced Interdisciplinary Research, Xidian University, Xi'an, Shaanxi, 710071, China.

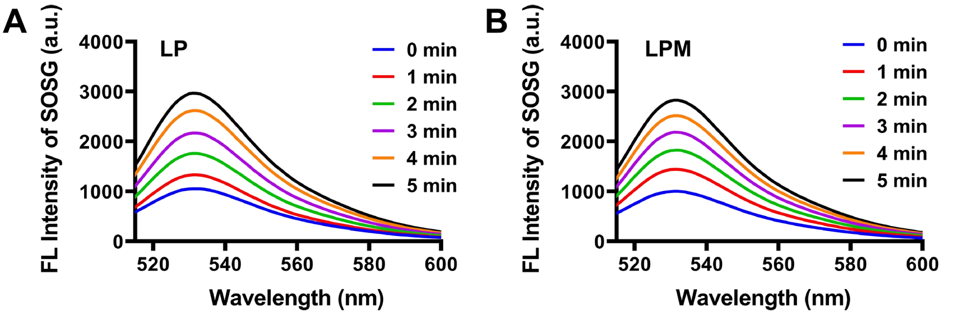

Figure S1. Fluorescence spectra of SOSG incubated with LP NPs (A) or LPM NPs (B) with ultrasound (US) irradiation for different time points (0-5 min).

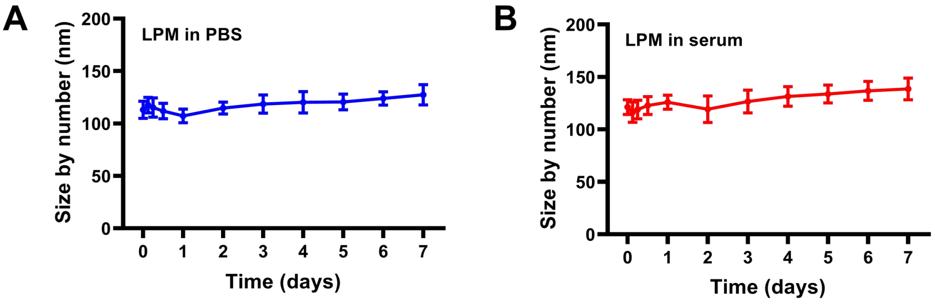

Figure S2. Stability of LPM NPs in PBS and serum. (A) Size changes of LPM NPs in PBS (pH 7.4) for 7 days. (B) Size changes of LPM NPs in serum for 7 days. The values are presented as the mean ± SD, n = 3.

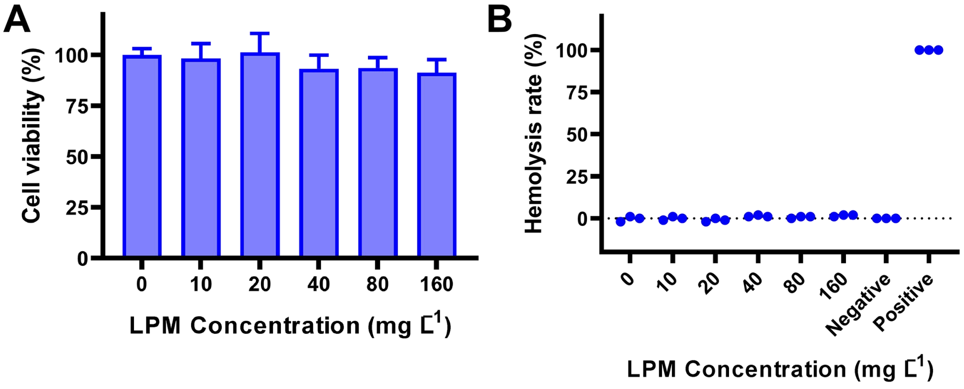

Figure S3. The MTT assay of NIH-3T3 cells after treated with (A) LPM at different concentrations for 24 h. The values are presented as the mean ± SD, n = 5. (B) Hemolysis rate after treated with different concentrations LPM.

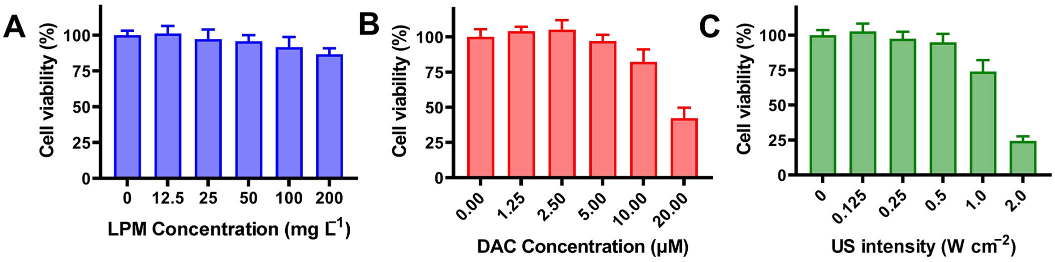

Figure S4. The MTT assay of 4T1 cells after treated with (A) LPM and (B) DAC at different concentrations for 24 h. (C) The MTT assay of 4T1 cells upon US irradiation with different US intensity for 5 min. The values are presented as the mean ± SD, n = 5.

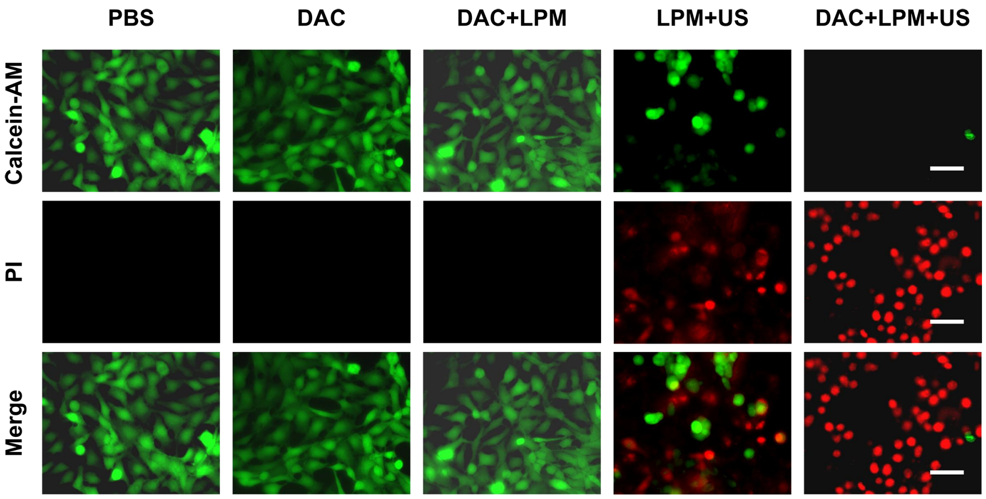

Figure S5. Living/Dead cell staining in 4T1 cells with different treatments. Cells incubated with calcein-AM (green fluorescence) and PI (red fluorescence) were immediately determined using an inverted fluorescence microscope. Scale bar: 50 μm.

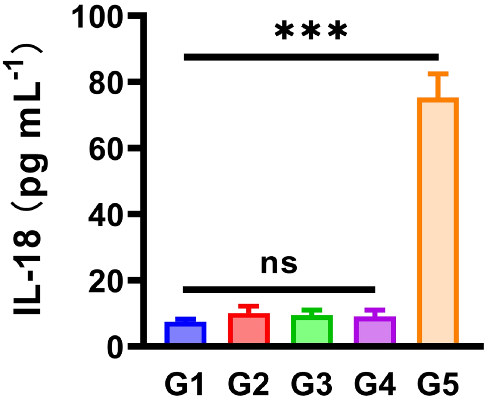

Figure S6. The release of IL-18 from 4T1 cells after different treatments. The differently treated groups are as follows: (G1) PBS, (G2) DAC, (G3) DAC + LPM, (G4) LPM under US irradiation, (G5) DAC + LPM under US irradiation. Data are presented as the mean ± SD, n=5. Statistical significance was calculated via unpaired t-test. (ns, nonsignificant; ***P < 0.001).

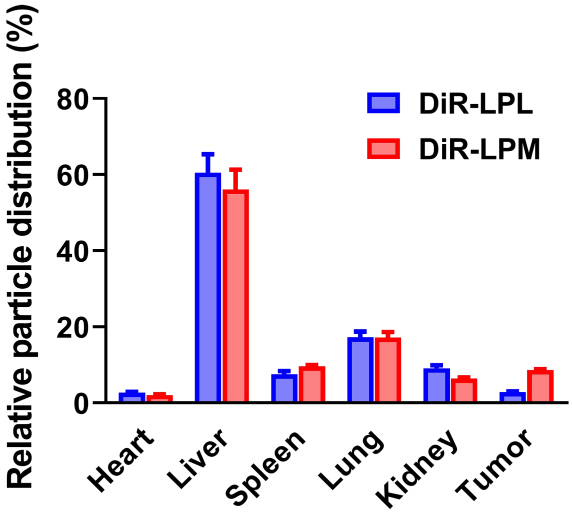

Figure S7. Relative distribution of DiR-LPL and DiR-LPM in the main organs and tumors of 4T1 tumor-bearing mice after intravenous injection for 24 h. The values are presented as the mean ± SD, n = 5.

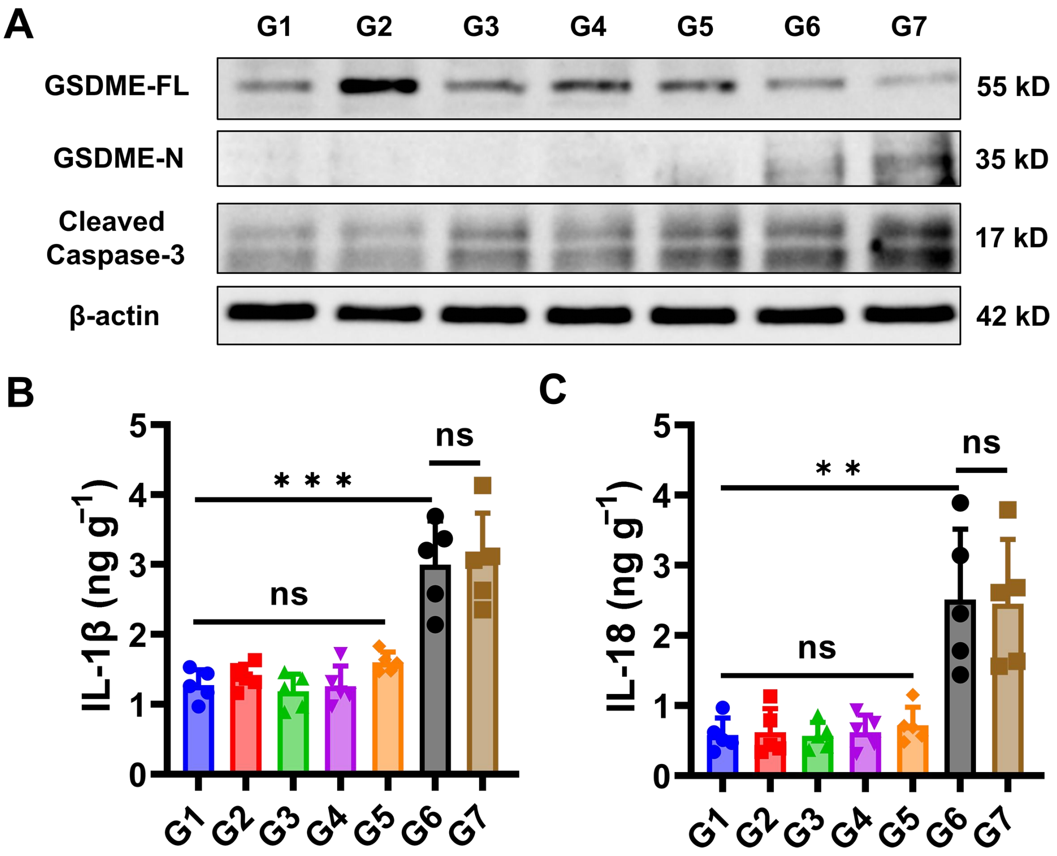

Figure S8. Western blot analysis (A) of GSDME-FL, GSDME-N and cleaved caspase-3 expression in the tumor tissue after different treatments. The release of IL-1β (B) and IL-18 (C) in the tumor homogenates after different treatments. The groups are as follows: (G1) PBS, (G2) DAC, (G3) PM, (G4) LPM, (G5) PM + US, (G6) DAC + PM + US and (G7) DAC + LPM + US. The values are presented as the mean ± SD, n = 5. Data are presented as the mean ± SD. Statistical significance was calculated via unpaired t-test. (ns, nonsignificant; ***P* < 0.01; ****P* < 0.001).

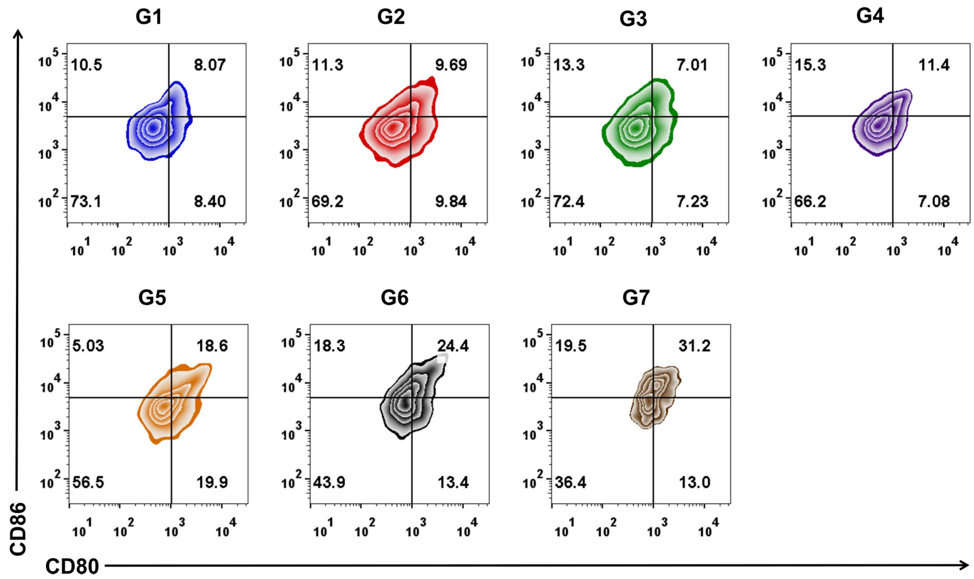

Figure S9. Representative flow cytometric analysis of CD80 and CD86 cells gating on CD11c+ cells in TDLNs after different treatment groups as follows: (G1) PBS, (G2) DAC, (G3) PM, (G4) LPM, (G5) PM + US, (G6) DAC + PM + US and (G7) DAC + LPM + US.

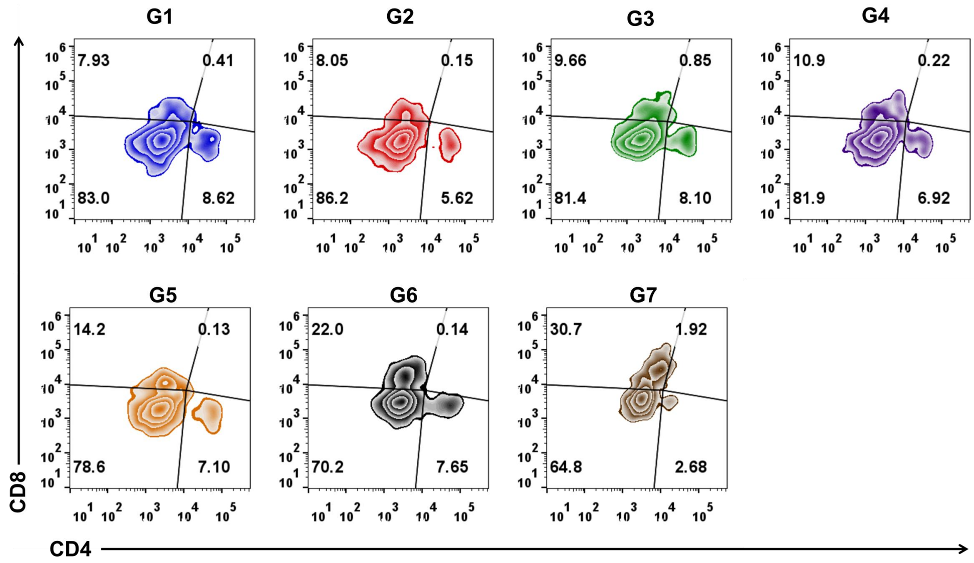

Figure S10. Representative flow cytometric analysis of CD8 and CD4 T cells gating on CD3+ T cells in tumors after different treatment groups as follows: (G1) PBS, (G2) DAC, (G3) PM, (G4) LPM, (G5) PM + US, (G6) DAC + PM + US and (G7) DAC + LPM + US.

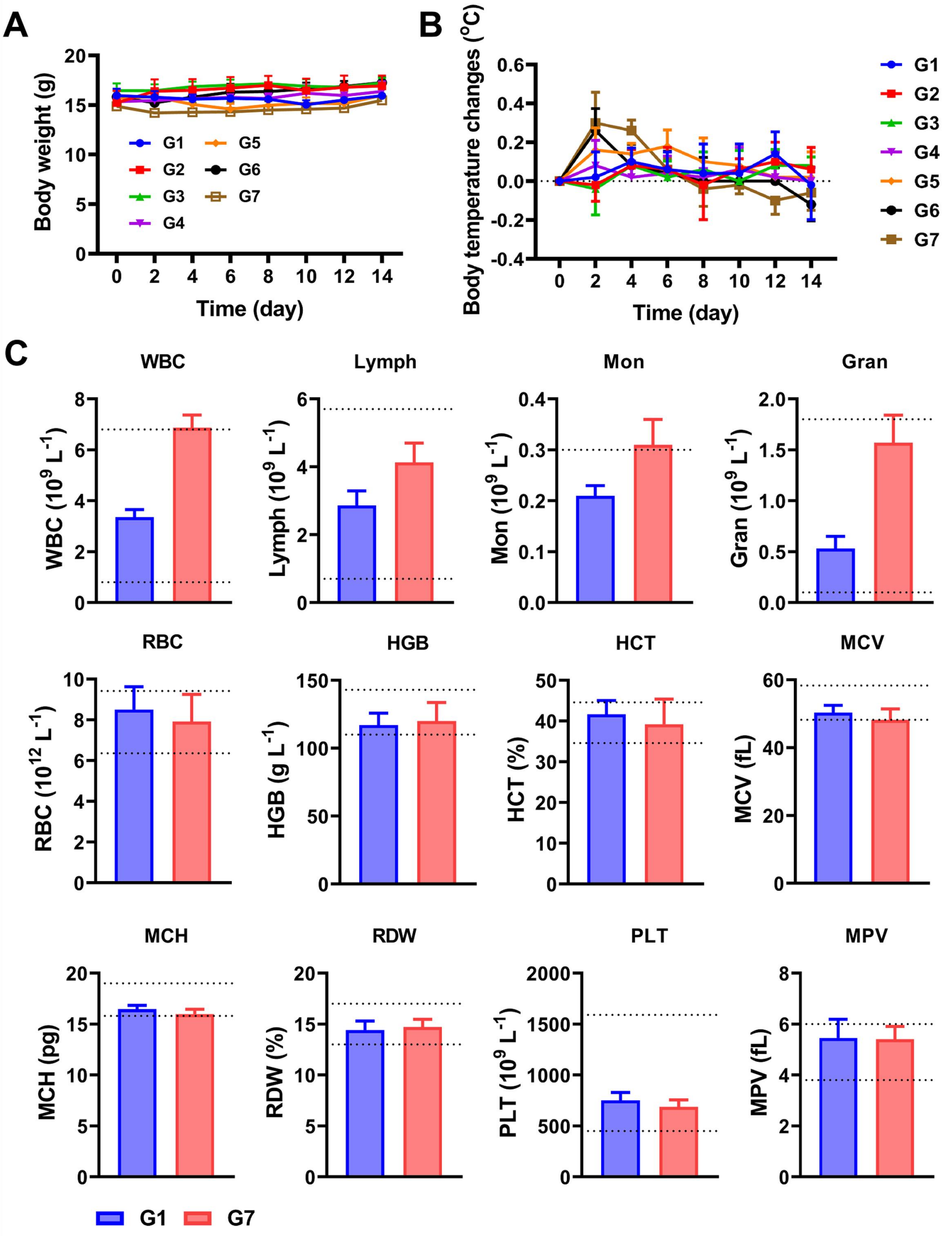

Figure S11. Changes of body weight (A) and body temperature (B) in mice treated with different groups. The groups are as follows: (G1) PBS, (G2) DAC, (G3) PM, (G4) LPM, (G5) PM + US, (G6) DAC + PM + US and (G7) DAC + LPM + US. The values are presented as the mean ± SD, n = 5. (C) Blood biochemical tests of BALB/c mice treated with G1 and G7. The values are presented as the mean ± SD, n = 3.

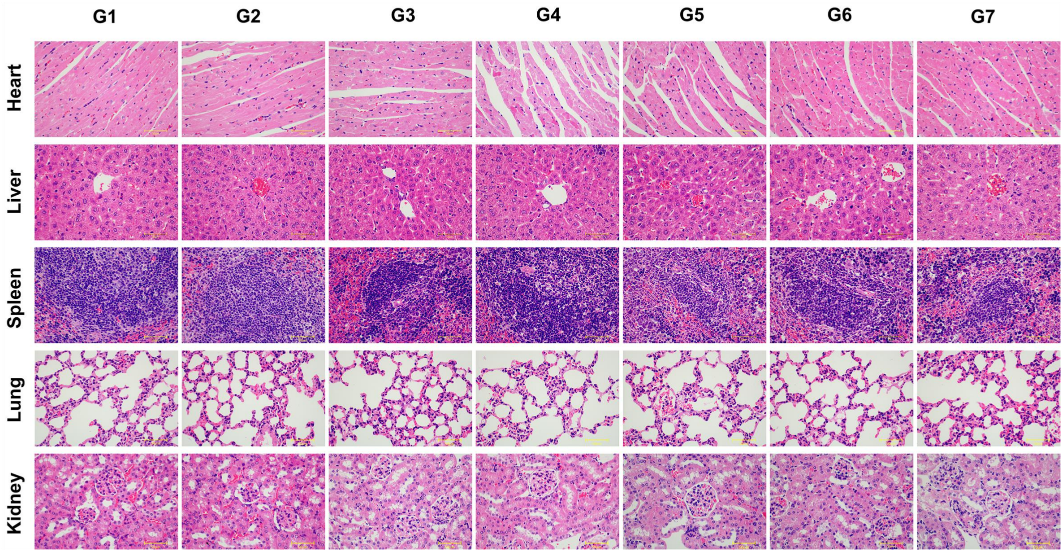

Figure S12. H&E staining of heart, liver, spleen, lung, and kidney in the mice from various groups. The groups are as follows: (G1) PBS, (G2) DAC, (G3) PM, (G4) LPM, (G5) PM + US, (G6) DAC + PM + US and (G7) DAC + LPM + US. Scale bar: 50 μm.
